# Accumulating Microglia Phagocytose Injured Neurons in Hippocampal Slice Cultures: Involvement of p38 MAP Kinase

**DOI:** 10.1371/journal.pone.0040813

**Published:** 2012-07-17

**Authors:** Takahiro Katayama, Hayato Kobayashi, Toshiyuki Okamura, Yuko Yamasaki-Katayama, Tatsuya Kibayashi, Hiroshi Kimura, Keiko Ohsawa, Shinichi Kohsaka, Masabumi Minami

**Affiliations:** 1 Department of Pharmacology, Graduate School of Pharmaceutical Sciences, Hokkaido University, Sapporo, Japan; 2 Department of Molecular Pharmacology, Graduate School of Pharmaceutical Sciences, Kyoto University, Kyoto, Japan; 3 Graduate School of Frontier Biosciences, Osaka University, Osaka, Japan; 4 Department of Neurochemistry, National Institute of Neuroscience, Tokyo, Japan; Chiba University Center for Forensic Mental Health, Japan

## Abstract

In this study, microglial migration and phagocytosis were examined in mouse organotypic hippocampal slice cultures, which were treated with *N*-methyl-D-aspartate (NMDA) to selectively injure neuronal cells. Microglial cells were visualized by the expression of enhanced green fluorescent protein. Daily observation revealed microglial accumulation in the pyramidal cell layer, which peaked 5 to 6 days after NMDA treatment. Time-lapse imaging showed that microglia migrated to the pyramidal cell layer from adjacent and/or remote areas. There was no difference in the number of proliferating microglia between control and NMDA-treated slices in both the pyramidal cell layer and stratum radiatum, suggesting that microglial accumulation in the injured areas is mainly due to microglial migration, not to proliferation. Time-lapse imaging also showed that the injured neurons, which were visualized by propidium iodide (PI), disappeared just after being surrounded by microglia. Daily observation revealed that the intensity of PI fluorescence gradually attenuated, and this attenuation was suppressed by pretreatment with clodronate, a microglia toxin. These findings suggest that accumulating microglia phagocytosed injured neurons, and that PI fluorescence could be a useful indicator for microglial phagocytosis. Using this advantage to examine microglial phagocytosis in living slice cultures, we investigated the involvements of mitogen-activated protein (MAP) kinases in microglial accumulation and phagocytosis. p38 MAP kinase inhibitor SB203580, but not MAP kinase/extracellular signal-regulated kinase inhibitor PD98059 or c-Jun *N*-terminal kinase inhibitor SP600125, suppressed the attenuation of PI fluorescence. On the other hand, microglial accumulation in the injured areas was not inhibited by any of these inhibitors. These data suggest that p38 MAP kinase plays an important role in microglial phagocytosis of injured neurons.

## Introduction

Microglia actively survey local brain environments by extending and retracting their processes in various directions in a normal brain [1]–[3]. In response to pathological alterations, microglia rapidly extend their processes toward the injured sites [4]. Following such rapid and short-term responses, microglial cells accumulate in the injured brain areas and are supposed to prevent expansion of damaged areas by engulfment and elimination of injured cells and cell debris. Research on microglial phagocytosis of injured cells, particularly injured neurons, has grown in recent years. The appearance of phosphatidylserine on the cell surface of apoptotic cells is known as an eat-me signal to trigger phagocytosis by microglia as well as macrophages [5]. Hirt and Leist [6] reported that phosphatidylserine is also translocated to the cell surface of necrotic cells and functions as an eat-me signal for phagocytosis by the murine microglia cell line BV-2. It was recently reported that TREM2 expressed on the cell surface of microglia is involved in microglial phagocytosis of injured neurons [7] and that HSP60 is one of the endogenous ligands for TREM2 [8]. However, these studies used dissociated cell cultures of primary microglia or microglia cell lines such as BV-2 and N9 to examine their phagocytic activity, although microglia *in vivo* migrate to and phagocytose injured neurons in the brain parenchyma where neural cells, including neurons and glial cells, and extracellular matrix are densely packed. To resolve this issue, we examined microglial migration and phagocytosis in organotypic hippocampal slice cultures that retained the cytoarchitecture of the original tissue to a large degree [9], [10]. In the present study, we first demonstrated microglial migration to the injured areas by using time-lapse imaging of the slice culture prepared from Iba1-EGFP mice. Next, we examined microglial phagocytosis of the injured neurons in slice cultures and revealed that the intensity of propidium iodide (PI) fluorescence could be a useful indicator of microglial phagocytosis. Using this advantage to examine microglial phagocytosis in living slice cultures, we then examined the effects of mitogen-activated protein (MAP) kinase inhibitors on microglial phagocytosis and revealed the crucial role of p38 MAP kinase in microglial phagocytosis of injured neurons.

## Materials and Methods

### Ethics Statements

All experimental procedures using animals were performed in accordance with the policies and recommendations of the NIH guidelines and with the approval of the Institutional Animal Experimentation Committee of Hokkaido University (Permit Number: 08-119) and Kyoto University Graduate School of Pharmaceutical Sciences (Permit Number: 2004-20).

### Reagents

*N*-methyl-D-aspartate (NMDA) was purchased from Nacalai Tesque (Kyoto, Japan). Clodronate was purchased from Sigma (St Louis, MO). PI was from Wako Pure Chemical (Osaka, Japan). SB203580 and PD98059 were from Calbiochem (San Diego, CA), and SP600125 was from Biomol (Plymouth, PA). All other reagents, unless otherwise indicated, were purchased from Nacalai Tesque.

### Culture Preparation

ddY mice (Japan SLC, Hamamatsu, Japan) and Iba1-EGFP transgenic mice derived from the C57BL/6 strain, microglial cells of which were visualized by the expression of EGFP [11], were used. Organotypic hippocampal slice cultures were prepared from 6- to 7-day-old male and female mice according to the standard interface method [12]–[14]. Briefly, mice were decapitated under aseptic conditions, their brains were removed, and hippocampi were rapidly dissected in ice-cold Hanks’ balanced salt solution (Invitrogen, Carlsbad, CA). The hippocampi were cut into 300-µm-thick slices using a tissue chopper (Narishige, Tokyo, Japan). They were transferred onto 30-mm Millicell-CM insert membranes (pore size, 0.4 µm; Millipore, Bedford, MA) that were placed in 6-well culture plates (BD Biosciences, San Jose, CA) or 35-mm glass-bottom culture dishes (Matsunami Glass Ind. Ltd., Kishiwada, Japan) with 1 mL of culture medium. Four to 6 slices were cultivated on each membrane at the interface between air and culture medium. The culture medium was composed of 50% HEPES-buffered minimum essential medium (Invitrogen), 25% Hanks’ balanced salt solution and 25% heat-inactivated horse serum (Invitrogen) supplemented with 6.5 mg/mL D-glucose, 2 mM L-glutamine, 100 U/mL penicillin G sodium, and 100 µg/mL streptomycin sulfate. The culture medium was replaced 2 days after the preparation and every 3 days thereafter. The slices were maintained at 37°C in a humidified atmosphere with 5% CO_2_ for 10–11 days before NMDA treatment.

### Treatment

To injure neuronal cells, slice cultures were treated with 50 µM NMDA for 3 h at 10 or 11 days *in vitro* (DIV). In the experiments using microglia-eliminated slice cultures, slice cultures were pretreated with 100 µM clodronate, a microglial toxin [15], [16], for 4–7 DIV. In the experiments to examine the involvement of MAP kinases in microglial migration and phagocytosis, MAP kinase inhibitors (1–10 µM) were added to culture medium 1 day after NMDA treatment.

### Immunohistochemistry

Slices were fixed with 4% paraformaldehyde in phosphate-buffered saline (PBS) containing 4% sucrose for 2 h at 4°C and stored in 25% sucrose at 4°C until use. For immunohistochemistry, slices were rinsed with PBS, blocked with 1.5% normal goat serum (Vector Laboratories, Burlingame, CA) in PBS containing 0.3% Triton X-100, and incubated with primary antibodies overnight at 4°C. For primary antibodies, rabbit anti-Iba1 antibody (2 µg/mL, #019-19741, Wako Pure Chemical) for microglia, mouse anti-NeuN antibody (5 µg/mL, #MAB377, Millipore) for neurons, mouse anti-glial fibrillary acidic protein (GFAP) antibody (1∶500, #G3893, Sigma) for astrocytes and rabbit anti-NG2 antibody (2 µg/mL, #AB5320, Millipore) for NG2-positive cells were used. The slices were rinsed with PBS and incubated with secondary antibodies for 1 h. For the secondary antibodies, Alexa Fluor 488-labeled goat anti-rabbit IgG antibody, Alexa Fluor 488-labeled goat anti-mouse IgG antibody and Alexa Fluor 568-labeled goat anti-mouse IgG antibody (6.7 µg/mL each; Invitrogen) were used. After rinsing in PBS, cultures were mounted on glass slides with VectaShield (Vector Laboratories). Immunofluorescent images were obtained with an inverted fluorescence microscope (IX-70; Olympus, Tokyo, Japan) equipped with a cooled CCD camera (VB-6010; KEYENCE, Osaka, Japan) or confocal laser-scanning microscopes (A1R; Nikon, Tokyo, Japan or LSM510; Carl Zeiss, Jana, Germany).

### Assessment of Microglial Accumulation

**Figure 1:**
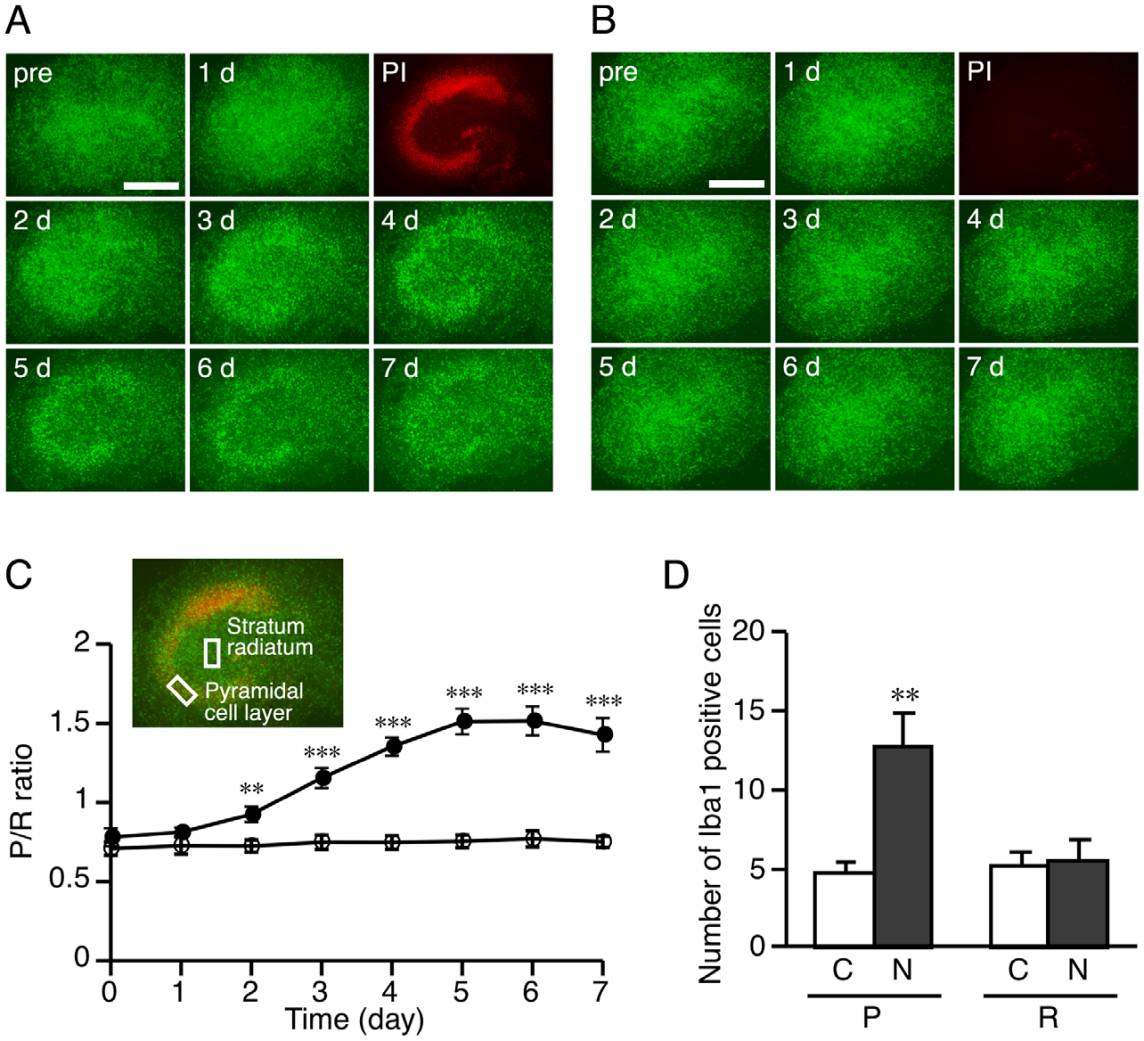
NMDA-induced neuronal injury caused microglial accumulation in the injured areas. **A, B:** Representative images of EGFP (green)-expressing microglia before (pre) or 1–7 days after treatment with NMDA (**A**) or vehicle (**B**). Injured cells at one day after the treatment are visualized by PI fluorescence (red). Scale bar = 500 µm. **C:** Accumulation of microglia in the injured areas was assessed by comparing the fluorescent intensity between the pyramidal cell layer and stratum radiatum. Fluorescent intensity in an area of 100 µm×200 µm (shown by white squares in the inset) was quantified by analyzing the captured fluorescent images using ImageJ software. The ratio of fluorescence intensity of the pyramidal cell layer (P) to that of the stratum radiatum (R) (designated as the P/R ratio) was calculated and used as an index of microglial accumulation in the injured areas. Microglial accumulation in the injured areas after treatment with NMDA (closed circle) or vehicle (open circle) was shown by the P/R ratio. Data are expressed as the mean ± SEM from seven slices. **P<0.01, ***P<0.001 versus pre (0 day) (one-way ANOVA, followed by Bonferroni *post hoc* test). **D:** The number of Iba1-immunopositive cells in the pyramidal cell layer (P) and stratum radiatum (R) at day 4 after NMDA treatment. Accumulation of microglia in the injured areas was assessed by counting the number of Iba1-immunopositive cells. Slice cultures at day 4 after treatment with vehicle (C) or NMDA (N) were subjected to immunostaining with anti-Iba1 antibody and then the number of Iba1-immunopositive cells in the areas of 200 µm×200 µm at three different z-positions (5-µm intervals) was counted. Data are expressed as the mean ± SEM from twelve areas of four slices. **P<0.01 versus pyramidal cell layers of vehicle-treated control slices (Welch’s *t*-test).

Slice cultures prepared from Iba1-EGFP transgenic mice were treated with NMDA. Neuronal injury induced by NMDA treatment was visualized by adding PI (0.5 µg/mL) to the culture medium. The culture medium was replaced with fresh medium containing PI after daily observation. PI-positive injured cells were observed especially in the pyramidal cell layer, accompanied by a slightly weaker PI staining in the dentate gyrus. Immunostaining with an anti-NeuN antibody showed that most of the PI-positive injured cells were neurons (data not shown). Fluorescent images for microglia (green) and injured cells (red) in each cultured slice were obtained daily before and 1–7 days after NMDA treatment using an inverted fluorescence microscope (IX-70; Olympus). Because EGFP fluorescence intensity was markedly increased throughout the whole slice cultures after neuronal injury probably because of the enhanced expression of Iba1 gene, the images for microglia (green) were obtained using optimal exposure time for each observation to clearly show the microglial cell distribution. Accumulation of microglia in the injured areas was assessed by comparing the fluorescent intensity between the pyramidal cell layer and stratum radiatum (Fig. 1C). Fluorescent intensity in an area 100 µm×200 µm was quantified by analyzing the captured fluorescent images using ImageJ software (version 1.40 g; NIH). The ratio of fluorescence intensity of the pyramidal cell layer (P) to that of the stratum radiatum (R) (designated as the P/R ratio) was calculated and used as an index of microglial accumulation in the injured areas.

In addition to the EGFP fluorescent image analysis, accumulation of microglia in the injured areas was assessed by counting the number of Iba1-immunopositive cells in the pyramidal cell layer and stratum radiatum at day 4 after NMDA treatment. Slice cultures at day 4 after treatment with vehicle or NMDA were subjected to immunostaining with anti-Iba1 antibody and then the number of Iba1-immunopositive cells in the areas of 200 µm×200 µm at three different z-positions (5-µm intervals) was counted. Data are expressed as the mean ± SEM from twelve areas of four slices.

### Detection of Proliferating Cells

Proliferating cells were detected by the incorporation of 5-ethynyl-2′-deoxyuridine (EdU) into DNA using the Click-iT EdU Assay Kit (Invitrogen). Slice cultures were incubated in EdU-containing medium for 48 h during 0–2 days or 2–4 days after NMDA or vehicle treatment, then fixed with 4% paraformaldehyde in PBS containing 4% sucrose for 2 h at 4°C. For detection of EdU-incorporated DNA, slices were washed in PBS containing 3% bovine serum albumin, permealized with 0.5% Triton X-100 in PBS and incubated with Alexa Fluor 568-labeled azide. After the detection procedure for EdU, immunostaining for cell-specific markers was carried out.

### Time-lapse Imaging

Time-lapse imaging for EGFP-expressing microglia (green) and PI-positive injured cells (red) in the slice culture was carried out over 60 h from day 3 after NMDA treatment using an inverted fluorescence microscope (IX-70; Olympus) with a 20× LUCPlanFL N (NA 0.45) objective lens. The cultured slices on a 30-mm Millicell-CM insert membrane placed in a 35-mm glass-bottom culture dish were put into the chamber mounted on the inverted fluorescence microscope and maintained at 37°C. A series of sets of fluorescent images were acquired at 20-min intervals and analyzed with a DeltaVision system (Applied Precision, Issaquah, WA). To make time-lapse movies, the acquired images were combined into time-lapse sequence by using MetaMorph software (Molecular Devices, Sunnyvale, CA).

### Measurement of PI-positive Areas

PI was added to the culture medium from 24 h before NMDA treatment. PI fluorescent images were acquired daily using an inverted fluorescence microscope (IX-70; Olympus) equipped with a cooled CCD camera (VB-6010; KEYENCE) 1–6 days after NMDA treatment. PI fluorescence images were obtained using a fixed exposure condition throughout the 7-day observation period for quantitative evaluation of neuronal injury. The culture medium was replaced with fresh medium containing PI after daily observation. The captured images were analyzed by ImageJ software. The intensity of PI fluorescence was binarized, and positive areas were determined in binary images and used as an index of the amount of injured cells.

### Statistical Analyses

Data are expressed as mean ± SEM. One-way analysis of variance followed by Bonferroni multiple comparison test, two-way ANOVA followed by Bonferroni multiple comparison test or Welch’s *t*-test in Prism 4.0 software (GraphPad Software, San Diego, CA) was used for statistical analyses. Differences with *P*<0.05 were considered statistically significant.

## Results

### Microglial Accumulation in the Pyramidal Cell Layer after NMDA Treatment

In the hippocampal slice cultures prepared from Iba1-EGFP transgenic mice, the distribution of microglial cells was observed daily for 7 days after NMDA treatment. Exposure to NMDA caused severe neuronal injury as shown by intense PI uptake (Fig. 1A). Microglial cells accumulated in the regions where marked PI uptake was observed, especially in the pyramidal cell layer (Fig. 1A). Quantitative analysis of the microglial accumulation by calculating the P/R ratio demonstrated that a significant accumulation started at day 2 after NMDA treatment and peaked at days 5 to 6 (Fig. 1C). No changes in the distribution of microglial cells over the 7-day observation period were observed in control slices (Fig. 1B, C). In addition to the EGFP fluorescent image analysis, the numbers of Iba1-immunoreactive cells within the pyramidal cell layer and stratum radiatum were counted at day 4 after NMDA treatment. The number of Iba1-positive cells in the pyramidal cell layer of NMDA-treated cultures was significantly increased compared with that of control cultures, while there was no difference in the number of Iba1-positive cells in the stratum radiatum between control and NMDA-treated groups (Fig. 1D).

### Microglial Proliferation and Migration in NMDA-treated Hippocampal Slices

**Figure 2:**
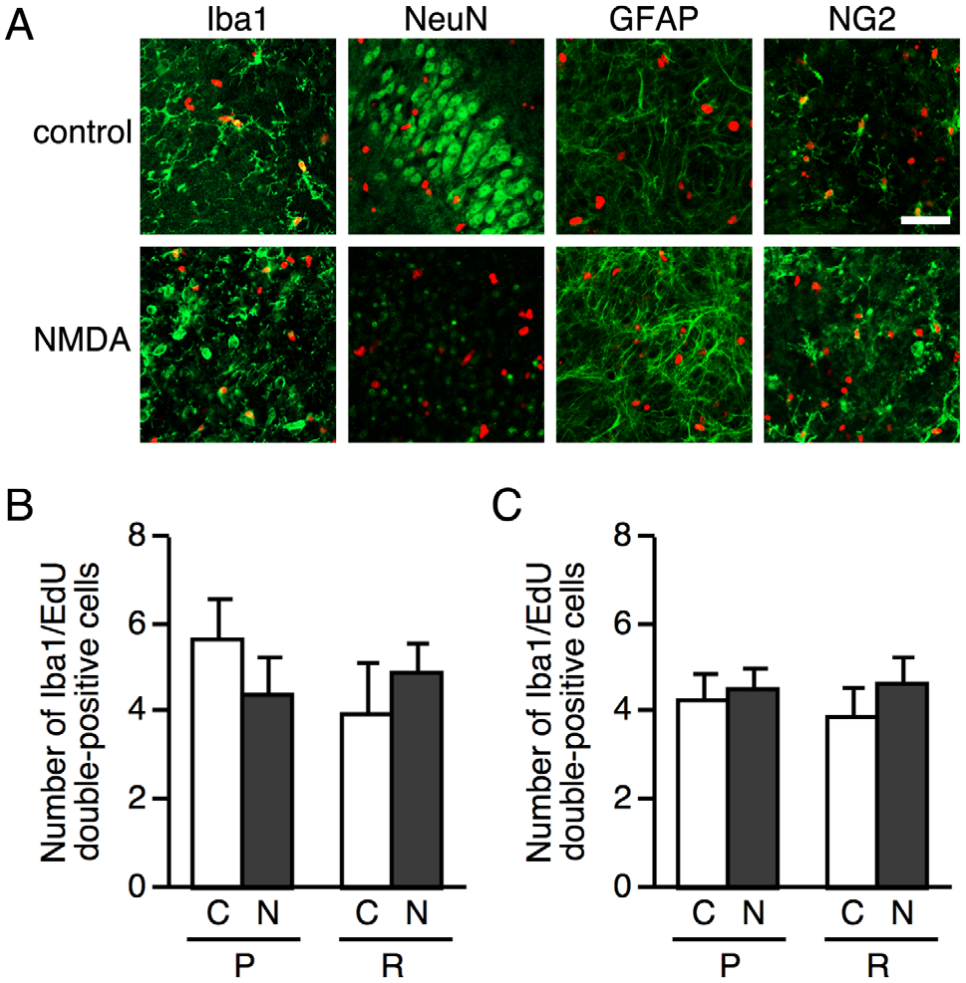
Proliferating cells after NMDA treatment. **A:** Representative confocal fluorescence images of EdU labeling (red) and cell-specific markers (green) in the pyramidal cell layer at 4 day after NMDA treatment. EdU (10 µM) was contained in the culture medium for 48 h during 2–4 days after NMDA treatment. After EdU detection, slice cultures were immunostained using the primary antibodies as follows: anti-Iba1 for microglia, anti-NeuN for neurons, anti-GFAP for astrocytes and anti-NG2 for NG2-positive cells. Scale bars = 50 µm. **B, C:** Iba1/EdU-double positive cells in the pyramidal cell layer (P) and stratum radiatum (R) of vehicle- or NMDA-treated slice cultures were counted. EdU was contained in the culture medium for 48 h during 0–2 days (**B**) or 2–4 days (**C**) after vehicle or NMDA treatment. Cell counting was carried out in the areas of 200 µm×200 µm at two different z-positions (6.9-µm intervals). C, vehicle; N, NMDA. Data are expressed as the mean ± SEM from twelve areas of six slices.

**Figure 3:**
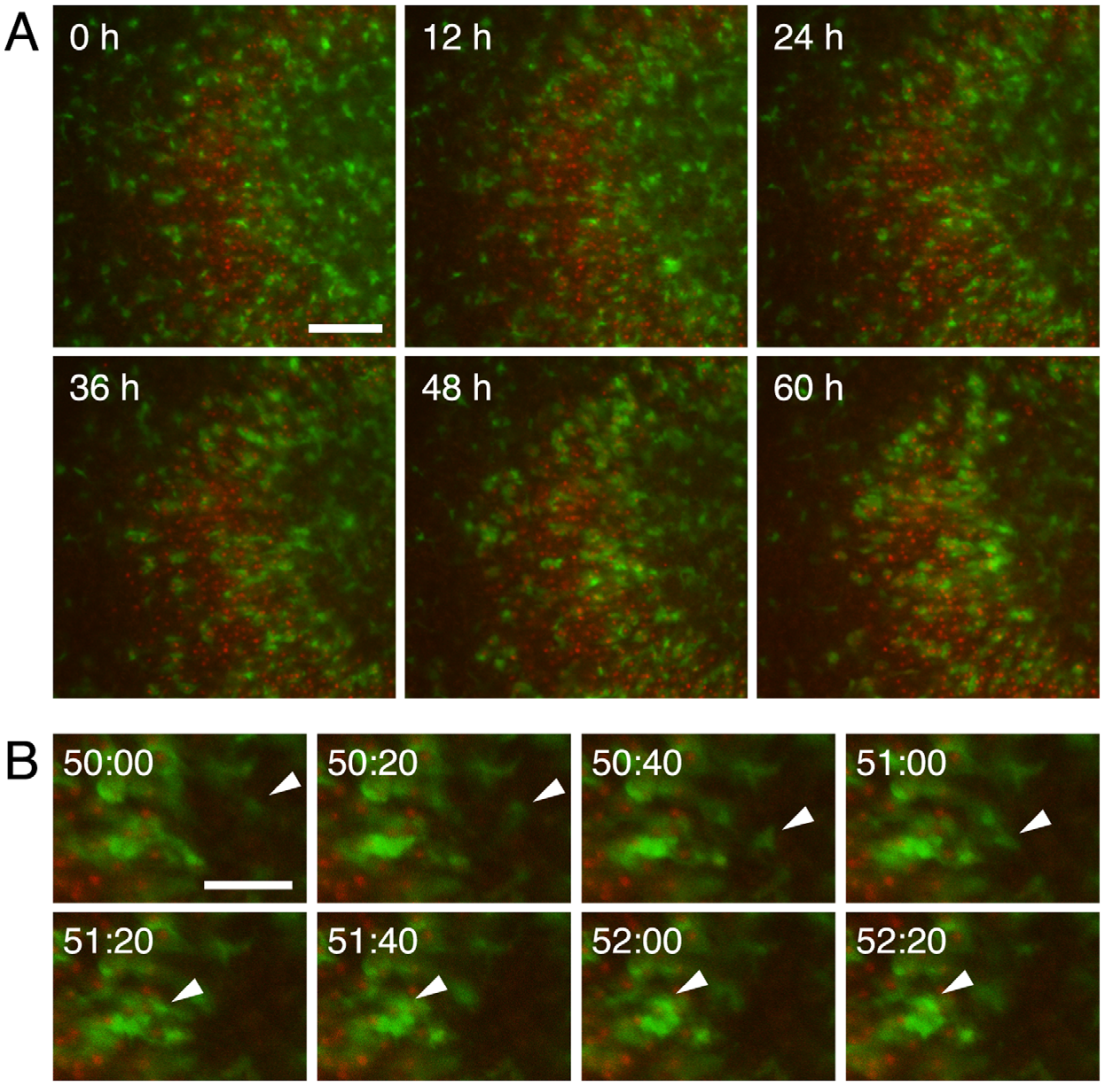
Time-lapse imaging for microglial migration to the injured areas after NMDA treatment. **A:** Time-lapse images (at 12-h intervals) of EGFP-expressing microglia (green) and PI-positive injured neurons (red) around the pyramidal cell layer for 60 h from day 3 after NMDA treatment. Scale bars = 100 µm. **B:** Time-lapse images (at 20-min intervals) of EGFP-expressing microglia (green) and PI-positive injured neurons (red) around the pyramidal cell layer from 50 h 00 min to 52 h 20 min after the beginning of time-lapse imaging. White arrowhead indicates migrating microglia toward the pyramidal cell layer. Scale bars = 50 µm.

To determine whether the accumulation of microglia in the injured areas was due to microglial proliferation, proliferating cells were visualized by the incorporation of EdU into DNA. EdU was contained in the culture medium for 48 h during 0–2 days or 2–4 days after NMDA or vehicle treatment, then visualization of EdU and immunohistochemistry were carried out at day 4, when the accumulation of microglia in the pyramidal cell layer was observed. In the pyramidal cell layer, the cells incorporating EdU during 2–4 days after the treatment were Iba1- or NG2-positive, but not NeuN- or GFAP-positive in both control and NMDA-treated slice cultures (Fig. 2A). Similar results were obtained when EdU was contained in the culture medium during 0–2 days (data not shown). There was no difference in the number of Iba1/EdU double-positive cells between control and NMDA-treated slices in both the pyramidal cell layer and stratum radiatum, whenever EdU was contained in the medium (Fig. 2B: 0–2 days, Fig. 2C: 2–4 days). These results suggest that the accumulation of microglia in the injured areas was not due to injured area-specific proliferation of microglia. Therefore, time-lapse imaging was then carried out for 60 h from day 3 after NMDA treatment to examine microglial migration in the slice cultures prepared from Iba1-EGFP transgenic mice. Time-lapse imaging showed that microglial cells migrated to the pyramidal cell layer from adjacent and/or remote areas (Fig. 3A, B and Movie S1).

### Microglial Phagocytosis of Injured Neurons after NMDA Treatment

**Figure 4:**
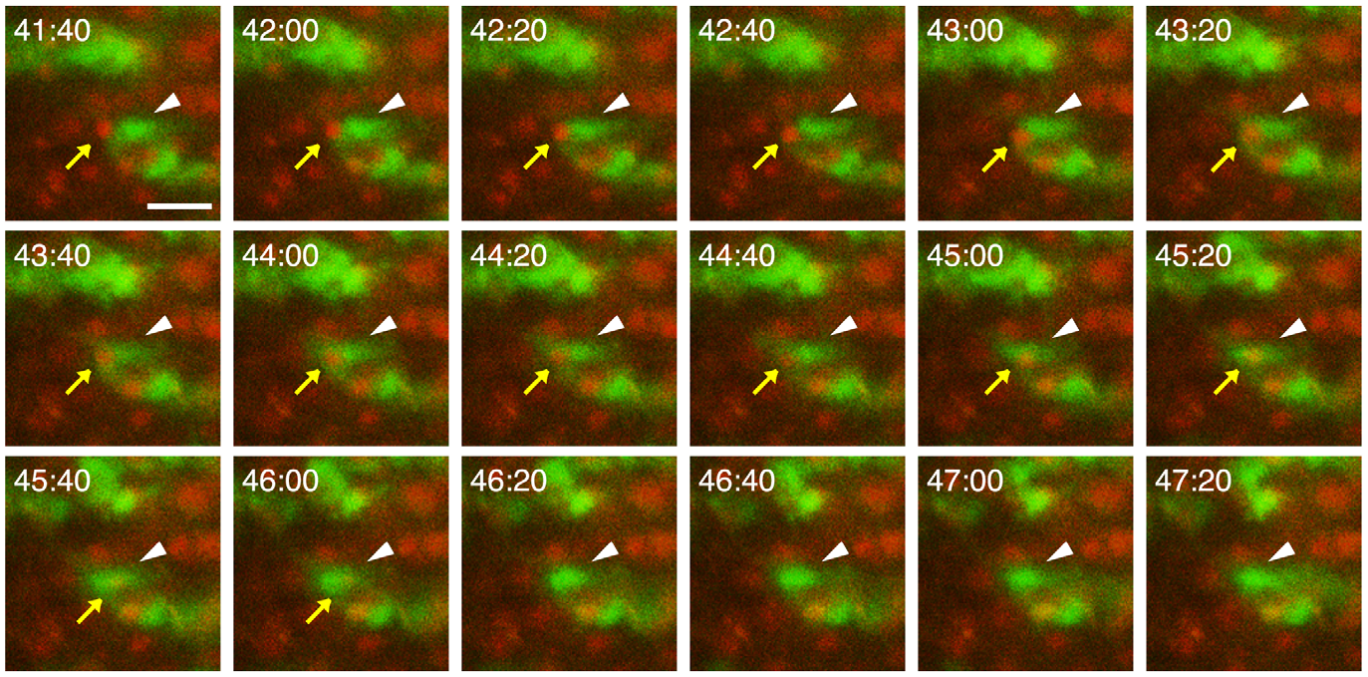
Time-lapse imaging for microglial phagocytosis of injured cells after NMDA treatment. Time-lapse images (at 20-min intervals) of EGFP-expressing microglia (green) and PI-positive injured neurons (red) in the pyramidal cell layer. White arrowhead and yellow arrow indicate the engulfing microglial cell and engulfed injured neuron, respectively. PI fluorescence of the injured neuron disappeared between 46 h 00 min and 46 h 20 min after the beginning of time-lapse imaging. Scale bars = 20 µm.

**Figure 5:**
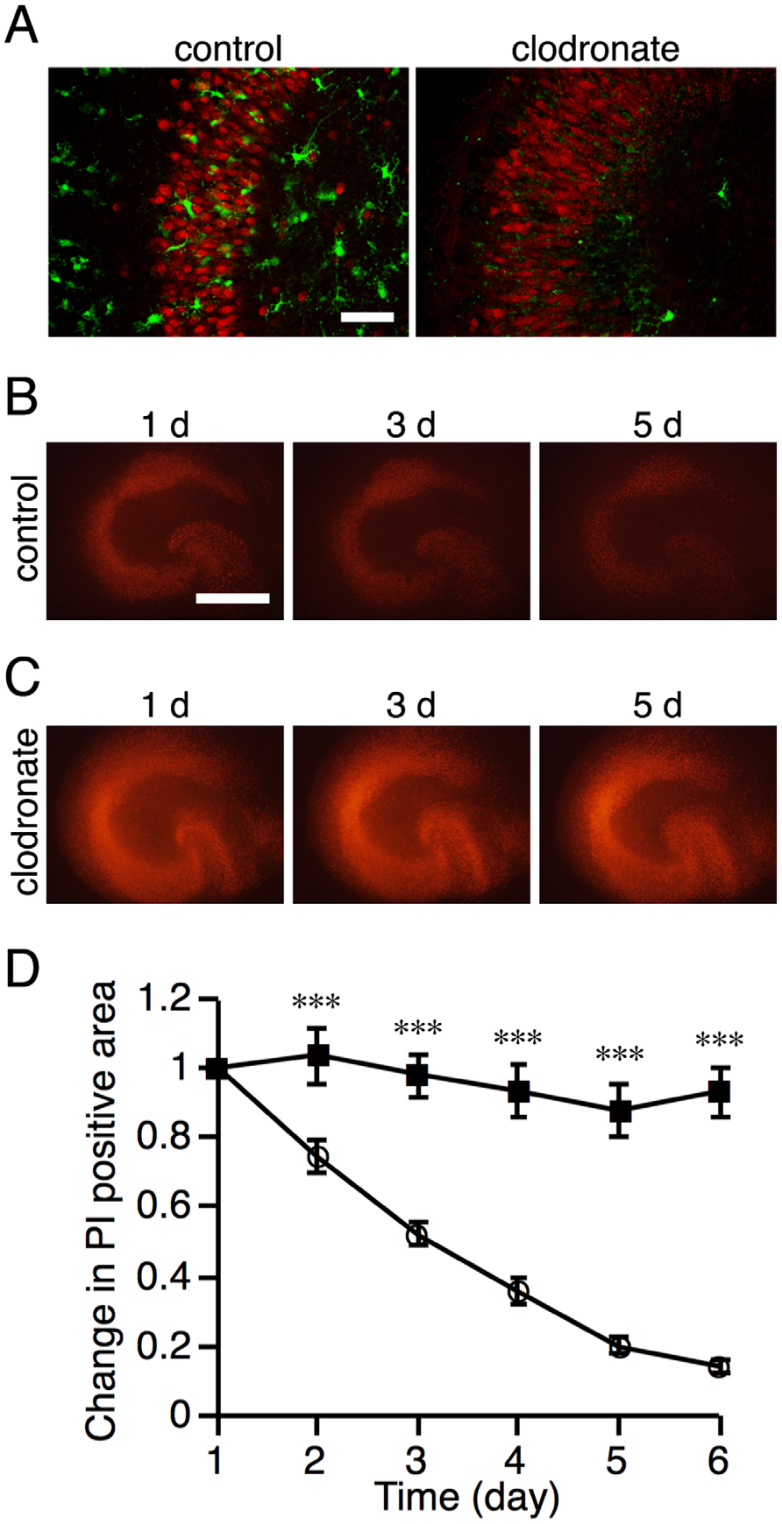
Time courses of the changes in PI fluorescence after NMDA treatment. **A:** Representative confocal images of double-labeled immunofluorescent staining for Iba1 (microglial marker, green) and NeuN (neuronal marker, red) of vehicle-pretreated (control) and clodronate-pretreated slice cultures at 10 DIV. The number of Iba1-immunopositive microglia in clodronate-pretreated cultures was markedly decreased compared with that in control cultures. Scale bars = 100 µm. **B, C:** Representative images of PI fluorescence at 1, 3 and 5 days after NMDA treatment in control (**B**) and clodronate-treated (**C**) slices. Scale bar = 500 µm. **D:** PI positive areas were measured daily for 6 days after NMDA treatment in control (open circle) or clodronate-treated (closed square) slices. Data are expressed as a ratio to the positive area at day 1. Data are expressed as the mean ± SEM from eleven control or nine clodronate-treated slices. ***P<0.001 versus control slices (two-way ANOVA, followed by Bonferroni *post hoc* test).

**Figure 6:**
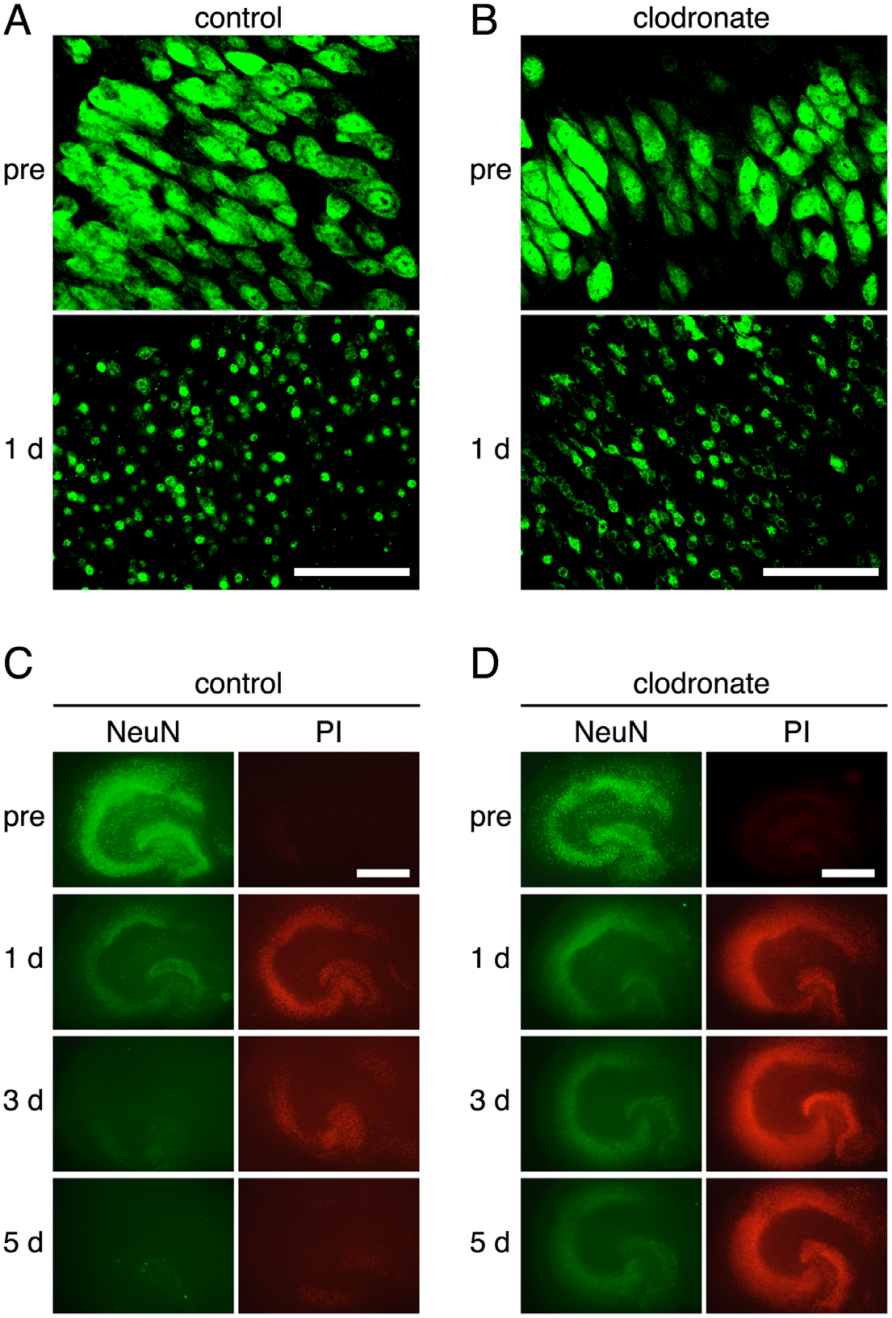
Time courses of the changes in NeuN immunoreactivity after NMDA treatment. **A, B:** Representative high magnification images of NeuN immunoreactivity before (pre) and 1 day after NMDA treatment in control (**A**) and clodronate-treated (**B**) slices. Treatment with clodronate did not affect neuronal viability (upper panels), and a similar extent of neuronal degeneration was observed between control and clodronate-treated slices 1 day after NMDA treatment (lower panels). Scale bar = 20 µm. **C, D:** Representative images of NeuN immunoreactivity and PI fluorescence before (pre) and 1, 3 and 5 days after NMDA treatment in control (**C**) and clodronate-treated (**D**) slices. Images of PI fluorescence were captured just before fixation for immunostaining of NeuN. Scale bar = 500 µm.

Time-lapse imaging in NMDA-treated slice cultures also revealed that PI-positive injured cells were surrounded by migrating microglia and then eliminated (Fig. 4 and Movie S2). This finding suggests the possibility that injured neurons are phagocytosed and eliminated by migrating microglia. To assess this possibility, we examined the effect of elimination of microglia by a pretreatment with clodronate on the elimination of injured cells. Immunostaining for the microglial marker Iba1 at 10 DIV confirmed that most microglial cells were eliminated by clodronate (Fig. 5A). There were no changes in the numbers and morphology of NeuN-positive cells (Fig. 5A and upper panels of Fig. 6A, B) and GFAP-positive cells (data not shown). Daily observation showed that the intensity of PI fluorescence after NMDA treatment was gradually attenuated in the control NMDA-treated slices (Fig. 5B, D), indicating the decrease in the number of injured cells. This attenuation of PI fluorescence was suppressed by pretreatment with clodronate (Fig. 5C, D). The same results were obtained in microglia-eliminated slice cultures by a pretreatment with liposome-encapsulated clodronate for 4–7 DIV (data not shown). These results suggest that the attenuation of PI fluorescence after NMDA treatment was due to microglial phagocytosis of injured neurons. However, an alternative possibility is that the elimination of microglial cells exaggerated neuronal injury and thereby enhanced PI fluorescence. Thus, we next examined the spatial and temporal patterns of NeuN immunoreactivity after NMDA treatment in control and microglia-eliminated (clodronate-pretreated) slices.

As above mentioned, pretreatment with clodronate did not affect the number or morphology of NeuN-positive neurons. After NMDA treatment, the morphology of NeuN staining clearly demonstrated the same degree of neuronal degeneration in control and clodronate-pretreated slices (Fig. 6A, B, lower panels). NeuN protein appeared to be left in the injured or dying neurons. NeuN immunoreactivity in control slices was gradually attenuated, and this attenuation showed a similar time course to that of PI fluorescence (Fig. 6C). The attenuation of NeuN immunoreactivity, as well as of PI fluorescence, was suppressed in clodronate-pretreated slices (Fig. 6D).

Collectively, these results indicate that injured/dying neurons were engulfed and eliminated by accumulating microglia and that PI fluorescence could be a useful indicator for microglial phagocytosis.

### Effects of MAP Kinase Inhibitors on Microglial Phagocytosis of Injured Neurons

**Figure 7:**
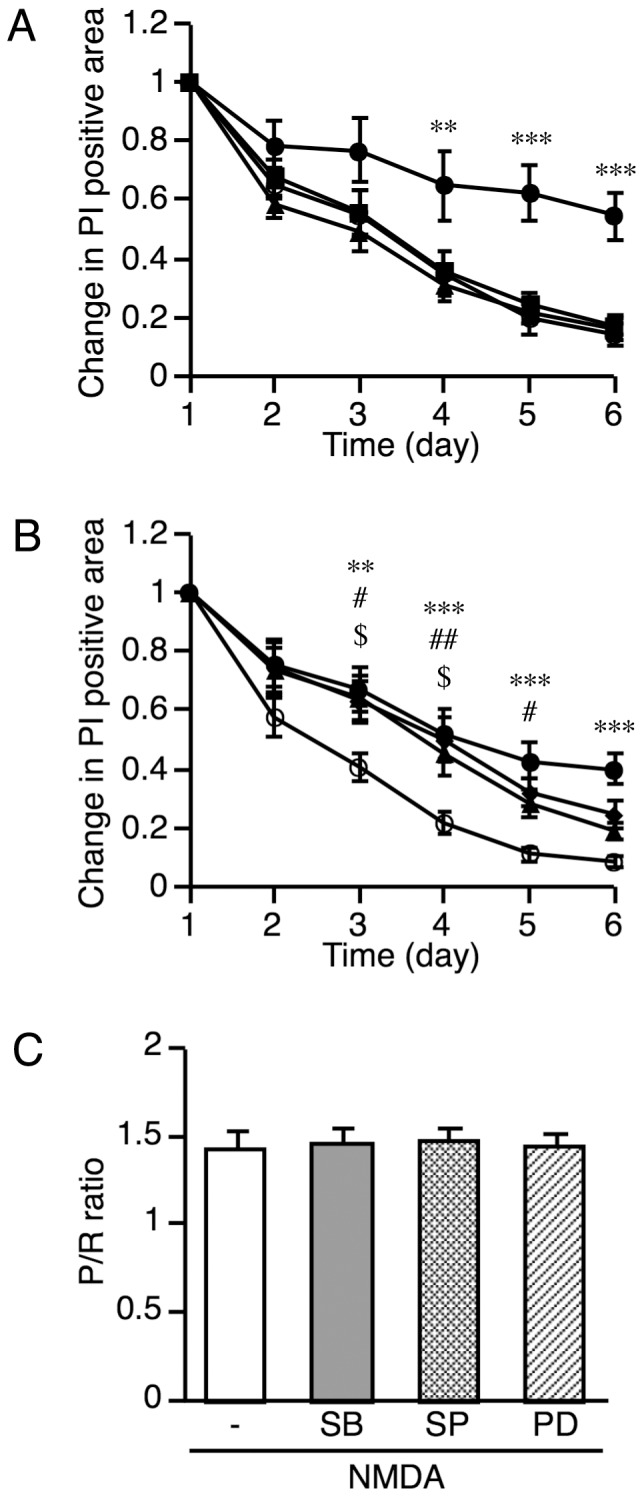
Effects of MAP kinase inhibitors on PI fluorescence and microglial accumulation after NMDA treatment. MAP kinase inhibitors were added to culture medium 1 day after NMDA treatment. **A:** Effects of MAP kinase inhibitors (10 µM each) on the attenuation of PI fluorescence after NMDA treatment. PI-positive areas were measured daily for 6 days after NMDA treatment in the presence of SB203580 (circle), SP600125 (triangle), PD98059 (diamond), or vehicle (square). Data are expressed as a ratio to the positive area at day 1. Data are expressed as the mean ± SEM from six to seven slices. **P<0.01, ***P<0.001 versus control slices (two-way ANOVA, followed by Bonferroni *post hoc* test). **B:** Concentration dependence of the suppressive effect of SB203580 on the attenuation of PI fluorescence after NMDA treatment. SB203580 at concentrations of 1 µM (closed triangle), 3 µM (closed diamond), and 10 µM (closed circle) were used. The open circle indicates the vehicle-treated group (control). Data are expressed as a ratio to the positive area at day 1. Data are expressed as the mean ± SEM from seven to eight slices. ^$^ *P*<0.05 between control and 1 µM SB203580, ^#^ *P*<0.05, ^##^ *P*<0.01 between control and 3 µM SB203580, **P<0.01, ***P<0.001 between control and 10 µM SB203580 (two-way ANOVA, followed by Bonferroni *post hoc* test). **C:** Microglial accumulation in the injured areas 6 days after NMDA treatment in the presence of MAP kinase inhibitors (10 µM each) was quantitatively analyzed by calculating the P/R ratio. SB, SB203580; SP, SP600125; PD, PD98059. Data are expressed as the mean ± SEM from three slices.

Using this advantage to examine microglial phagocytosis in living slice cultures by monitoring PI fluorescence, we investigated the involvement of MAP kinases in microglial phagocytosis. To avoid the influence of MAP kinase inhibitors on NMDA-induced neuronal injury, the inhibitors were added 1 day after NMDA treatment. The attenuation of PI fluorescence was significantly suppressed by the p38 MAP kinase inhibitor SB203580, but not by the MAP kinase/extracellular signal-regulated kinase inhibitor PD98059 or the c-Jun *N*-terminal kinase inhibitor SP600125 (Fig. 7A). The effect of SB203580 was concentration dependent (Fig. 7B). To clarify whether this suppressive effect of SB203580 was due to the inhibition of microglial phagocytosis of injured neurons or to the inhibition of microglial migration to the injured areas, the effect of SB203580 on microglial migration to the injured areas was examined by calculating the P/R ratio in Iba1-immunostained slices at 6 days after NMDA treatment. The result showed that none of the MAP kinase inhibitors, including SB203580, affected microglial migration to the injured areas (Fig. 7C), suggesting that the suppressive effect of SB203580 on the attenuation of PI fluorescence was due to the inhibition of microglial phagocytosis of injured neurons.

## Discussion

In the present study using hippocampal slice cultures, we demonstrated that NMDA-induced neuronal injury caused microglial accumulation in the injured areas. This result was consistent with the previous report [17], which demonstrated migration of microglial cells to the injured areas by combining hippocampal slice cultures with fluorescence-labeled microglial dissociated cultures. In this study, time-lapse imaging using hippocampal slice cultures prepared from Iba1-EGFP transgenic mice clearly demonstrated the migration of intrinsic microglial cells to the injured pyramidal cell layer. Together with the results that there was no difference in the number of Iba1/EdU double-positive cells between control and NMDA-treated slices in both the pyramidal cell layer and stratum radiatum, the findings suggest that the microglial accumulation in the injured brain areas was due to microglial migration from adjacent and/or remote areas rather than to microglial proliferation at the injured sites. We showed the increment of the number of microglial cells in the pyramidal cell layer by cell counting. However, there was no difference in the number of microglial cells in the stratum radiatum between control and NMDA-treated groups. One possible reason for no decrement of microglial cells in the stratum radiatum is the enhanced expression of Iba1 after neuronal injury, which may lead to the apparent increase in the number of Iba1-immunoreactive cells.

Time-lapse imaging also revealed that PI-positive neurons, which had been injured by NMDA treatment, were surrounded by migrating microglia and then eliminated. Similar findings were reported by Petersen and Dailey [18]. Using time-lapse imaging of fluorescent (green)-labeled microglia, they showed that fluorescent (red)-labeled damaged cells, which had been mechanically injured by the slicing procedure, were engulfed by migrating microglia. In the present study, we additionally demonstrated by daily observation that PI fluorescence after NMDA treatment was gradually attenuated and this attenuation was suppressed by elimination of microglia by pretreatment with clodronate during 4 to 7 DIV. This finding suggested two possibilities: the attenuation of PI fluorescence was due to microglial phagocytosis of injured neurons, or the elimination of microglial cells exaggerated neuronal injury and thereby enhanced PI fluorescence. The results from the examination of spatial and temporal patterns of NeuN-immunoreactivity after NMDA treatment in control and microglia-eliminated slices supported the former possibility. That is, NeuN immunoreactivity in the degenerated neurons was gradually attenuated over a time course similar to that of attenuation of PI fluorescence, and the attenuation of NeuN immunoreactivity, as well as of PI fluorescence, was suppressed in microglia-eliminated slices.

Importantly, the present results demonstrated that the intensity of PI fluorescence was not only positively correlated with the degree of cell injury, but was also negatively correlated with that of microglial phagocytosis. This means that examination by PI fluorescence only is insufficient to evaluate the degree of cell injury in preparations containing phagocytic cells, such as microglia, although PI fluorescence has been widely used for evaluation of cell injury. In such preparations, additional assessments, such as counting viable cells and measuring lactate dehydrogenase release, are necessary to evaluate cell injury. In addition, the present findings suggest that PI fluorescence can be used to monitor microglial phagocytosis in living slice cultures, whereas the degree of cell injury in such preparations should be checked by other appropriate methods such as NeuN staining used in this study.

SB203580, a p38 MAP kinase inhibitor, suppressed the attenuation of PI fluorescence after NMDA-induced neuronal injury in a concentration-dependent manner, suggesting two possibilities: the inhibition of microglial phagocytosis by SB203580 or the inhibition of microglial migration to the injured areas. Because the present results revealed no effect of SB203580 on microglial accumulation in the injured areas, it is likely that SB203580 suppressed the attenuation of PI fluorescence by inhibiting microglial phagocytosis of, but not microglial migration to, the injured neurons. Tanaka et al. [19] demonstrated that p38 MAP kinase is required for microglial phagocytosis of degenerated axon debris. The present results demonstrated that p38 MAP kinase is also involved in phagocytosis of injured cell bodies by intrinsic microglia in organotypic hippocampal slice cultures.

Some recent studies have suggested candidates for signal molecules, which are released/leaked from injured cells and activate microglial phagocytosis. Koizumi et al. [20] reported that extracellular UTP/UDP triggered microglial phagocytosis through P2Y6 receptors. Hsieh et al. [7] demonstrated that TREM2 and its ligand play an important role in microglial phagocytosis of apoptotic neurons. More recently, Fuhrmann et al. [21] demonstrated the involvement of microglial CX_3_CR1 and its ligand fractalkine in microglial phagocytosis of injured neurons in the brains of Alzheimer’s disease model mice. However, extracellular signaling from injured cells to microglia to regulate phagocytic processes remains to be elucidated. In the present study, we demonstrated that PI fluorescence is a useful indicator to monitor microglial phagocytosis of injured neurons in living slice cultures, especially after the severe insults that degenerate most of the neurons as observed in this study. Examination of microglial phagocytosis in living slice cultures may be useful for further elucidation of the underlying mechanisms of microglial phagocytosis regulation.

## Supporting Information

Movie S1 **Time-lapse movie of microglial migration to the injured area after NMDA treatment.** Fluorescent images of EGFP-expressing microglia (green) and PI-positive injured neurons (red) were acquired at 20-min intervals for 60 h from day 3 after NMDA treatment around the pyramidal cell layer in slice cultures prepared from Iba1-EGFP transgenic mice. Acquired images were converted to a time-lapse movie using MetaMorph software (Molecular Devices). The images picked up at 12-h intervals are shown in Figure 3A.
(MP4)

Movie S2 **Time-lapse movie of microglial phagocytosis of injured cells after NMDA treatment.** Fluorescent images of EGFP-expressing microglia (green) and PI-positive injured neurons (red) were acquired at 20-min intervals in the pyramidal cell layer of slice cultures prepared from Iba1-EGFP transgenic mice from 41 h 40 min to 47 h 20 min after the beginning of time-lapse imaging. Acquired images were converted to a time-lapse movie using MetaMorph software (Molecular Devices). Individual sequential images are shown in Figure 4.
(MP4)
